# Selective cell cycle arrest and induction of apoptosis in human prostate cancer cells by a polyphenol-rich extract of *Solanum nigrum*

**DOI:** 10.3892/ijmm.2011.835

**Published:** 2011-11-10

**Authors:** AKBAR NAWAB, VIJAY S. THAKUR, MOHAMMAD YUNUS, ABBAS ALI MAHDI, SANJAY GUPTA

**Affiliations:** 1 Department of Urology, Case Western Reserve University, Cleveland, OH 44106, USA; 2 The Urology Institute, University Hospitals Case Medical Center, Cleveland, OH 44106, USA; 3 Case Comprehensive Cancer Center, Cleveland, OH 44106, USA; 4 Department of Environmental Sciences, Babasaheb Bhimrao Ambedkar University, Lucknow 226025, India; 5 Department of Biochemistry, King George’s Medical University, Lucknow 226003, India

**Keywords:** apoptosis, DNA fragmentation, cell proliferation, prostate cancer, chemotherapy

## Abstract

Progression of prostate cancer is associated with escape of tumor cells from cell cycle arrest and apoptosis. Agents capable of selectively eliminating cancer cells by cell cycle arrest and/or induction of apoptosis offer a highly desirable approach. Here we demonstrate that a polyphenolic extract derived from ripe berries of *Solanum nigrum* (SN) differentially causes cell cycle arrest and apoptosis in various human prostate cancer cells without affecting normal prostate epithelial cells. Virally transformed normal human prostate epithelial PZ-HPV-7 cells and their cancer counterpart CA-HPV-10 cells, were used to evaluate the growth-inhibitory effects of the SN extract. SN treatment (5–20 μg/ml) of PZ-HPV-7 cells resulted in growth inhibitory responses of low magnitude. In sharp contrast, SN treatment of CA-HPV-10 cells increased cytotoxicity, decreased cell viability and induced apoptosis. Similar results were noted in the human prostate cancer LNCaP, 22Rv1, DU145 and PC-3 cell lines, where significant reductions in cell viability and induction of apoptosis was observed in all these cells, an effect independent of disease stage and androgen association. Cell cycle analysis revealed that SN treatment (5–20 μg/ml) resulted in a dose-dependent G2/M phase arrest and subG1 accumulation in the CA-HPV-10 but not in the PZ-HPV-7 cell line. Our results, for the first time, demonstrate that the SN extract is capable of selectively inhibiting cellular proliferation and accelerating apoptotic events in prostate cancer cells. SN may be developed as a promising therapeutic and/or preventive agent against prostate cancer.

## Introduction

A rational approach to cancer treatment is to selectively eliminate proliferating tumor cells via programmed cell death and spare quiescent or terminally differentiated cells (1,2). Decreasing the rate of programmed cell death or apoptosis in defective cells and increasing that of the cell cycle affords cancer cells a survival advantage and the ability to sustain and proliferate (2). In the United States, prostate cancer is highly prevalent and remains the second leading cause of cancer-related deaths among men (3). The major cause of mortality from this disease is metastasis of androgen-refractory cancer cells that fail to respond to hormone ablation therapy (4). As surgery and current chemotherapeutic options seem to be inadequate in curing or controlling prostate cancer, there is a pressing need for identification of novel agents for the management of this disease. Natural phytochemicals derived from dietary sources or medicinal plants have gained significant recognition in the control and containment of carcinogenesis and are considered a practical approach in disease prevention and therapy (5).

*Solanum nigrum* L. (Solanaceae) or ‘Black nightshade’ is an herbal plant indigenous to Southeast Asia and is commonly used as traditional folk medicine believed to possess promising biological activity (6). Certain parts of the plant have been used as a hepatoprotective agent to cure inflammation and edema (7,8). The water extracts of *Solanum nigrum* (SN) have been shown to exert cytoprotection against gentamicin-induced toxicity in Vero cells, suppress oxidant-mediated DNA damage and induce necrosis in SC-M1 stomach cancer cells (9,10). The SN extract has been shown to inhibit 12-O-tetradecanoylphorbol-13-acetate-induced tumor promotion in MCF-7 cells and to have anti-neoplastic activity against sarcoma in mice (11). It has also been reported that an ethanolic extract from fruits of SN could inhibit proliferation of human MCF-7 breast cancer cells, and induce cell death by apoptosis (11,12). Recent studies demonstrate that an ethanolic extract of SN was protective against chemical-induced hepatic injury and early hepatocarcinogenesis through overexpression of glutathione S-transferases and other phase II antioxidant enzymes (13). Extracts from whole plants of SN have been reported to result in hepatoma cell death by inducing autophagy and apoptosis and to inhibit cell growth of HepG2 hepatocarcinoma cells by inducing G2/M phase cell cycle arrest (14). These studies suggest that SN could exert its anti-neoplastic activity as a cancer preventive and therapeutic agent. However, there is limited information about whether SN exerts selective toxicity to cancer cells with minimal damage to normal cells, an effective strategy for eliminating cancer cells. This study provides the first evidence that aqueous polyphenolic-rich SN extract at microgram concentrations imparts differential anti-proliferative and apoptotic effects in human prostate carcinoma cells vs. non-cancerous cells.

## Materials and methods

### Preparation of Solanum nigrum extract

The ripe berries of *Solanum nigrum* L. were purchased from The State Unani Tibbya College, Lucknow, India. The plants were authenticated by the Department of Pharmacognosy, National Botanical Research Institute, Lucknow, India, where the voucher specimen has been deposited. The ripe berries were weighed and crushed to powder with a mortar and pestle, and a 5% (w/v) suspension was prepared in a flask by adding distilled hot boiling water. The flask was then placed on a shaker (200 rpm) for 4 h, and the temperature was maintained at 37°C. Subsequently, the flask was brought to room temperature and then the suspension was filtered through a series of Whatman filters and finally passed through a 0.22 μm filter (Millipore, Billerica, MA). The filtered aqueous extract was lyophilized on a Speed Vac and the residue was used for further experiments.

### Cell culture

The virally transformed PZ-HPV-7 cells derived from normal tissue of the peripheral zone of the prostate and immortalized by transfection with the HPV-18 virus as well as their cancer counterpart CA-HPV-10 cells and the other human prostate cancer cells, 22Rv1, LNCaP, DU145 and PC-3, were obtained from the American Type Culture Collection (Manassas, VA). All cells were cultured in appropriate culture medium at 37°C in a humidified atmosphere of 5% CO_2_.

### Composition analysis

The polyphenolic content present in the SN extract was analyzed according to the Folin-Ciocalteu method as previously described (15). Briefly, 0.1 mg of lyophilized material was dissolved in 1 ml distilled water and the solution was scanned from 200–750 nm on FluoStar Omega Spectrophotometer (BMG Labtech) and then subjected to absorbance at 750 nm using gallic acid as a standard. In the next step, aqueous SN extract was analyzed on an Agilent 1200 HPLC system (Agilent Technologies, Santa Clara, CA) using a C 18 column. The mobile phase consisted of acetonitrile and water as the isocratic solvent (30:70, v/v) maintained at a flow rate of 1 ml/min with an injection volume of 5 μl and a run time of 8 min as previously described (16). The resulting aqueous SN extract was loaded onto a 300 μm ID × 5 mm C18, PepMap nano reverse phase (RP) trapping column to pre-concentrate using UltiMate-3000 (Dionex, Sunnyvale, CA). The sample was further analyzed on a 75 μm ID × 15 cm C18, PepMap reverse phase nano separation column using the nano separation platform UltiMate-3000. Extract mixtures eluted from the column with the final gradient of acetonitrile of 20–80% for 30 min by mixing solvents A (100% water, 0.1% formic acid) and B (80% acitonitrile, 20% water, 0.1% formic acid) were introduced into an LTQ FT mass spectrometer (ThermoFisher Scientific, San Jose, CA). The instrument was equipped with a nano spray ion source with a needle voltage of 2.4 kV. MS and tandem MS spectra were acquired in the positive ion mode, with the following acquisition cycle: a full scan recorded in the FT analyzer at resolution R=100,000 followed by MS/MS of the eight most-intense ions in the LTQ analyzer.

### Proliferation assay

The effect of the SN extract on cell proliferation was determined by the MTT [3-(4,5-dimethylthiazol-2-yl)-2, 5-diphenyl tetrazoliumbromide] assay and the growth inhibition was assessed as the percent viability where vehicle-treated cells were taken as 100% viable as previously described (16).

### Light microscopy

PZ-HPV-7 and CA-HPV-10 cells were grown to 70% confluence and treated with 10–20 μg/ml concentrations of SN extract for 24 h. The photographs were captured at ×40 magnification using a phase-contrast inverse microscope (Olympus, Japan).

### DNA fragmentation assay

The cells were grown to ~70% confluence and treated with 5–20 μg/ml SN extracts for 48 h. Cells were then subjected to processing for DNA isolation and fragmentation assay. The bands were visualized under a UV transilluminator, followed by digital photography as previously described (16).

### TUNEL assay

The TUNEL assay for quantification of apoptosis was performed in PZ-HPV-7 and CA-HPV-10 cells after treatment with a 5–20 μg/ml concentration of the SN extract for 48 h using the APO-BRDU apoptosis kit (Phoenix Flow Systems, San Diego, CA) according to the manufacturer’s protocol using the EPICS-XL MCL flow cytometer.

### Flow cytometry

Cell cycle analysis was performed in PZ-HPV-7 and CA-HPV-10 cells after treatment with 5–20 μg/ml concentration of SN extract for 24 h using the EPICS-XL MCL flow cytometer and the Cell Quest analysis ModFit software as previously described (16).

### Statistical analysis

The values are expressed as means ± SD. The differences between the control and treated groups was determined by the Student’s t-test, and P-values <0.05 were taken to denote significant differences.

## Results

**Figure 1:**
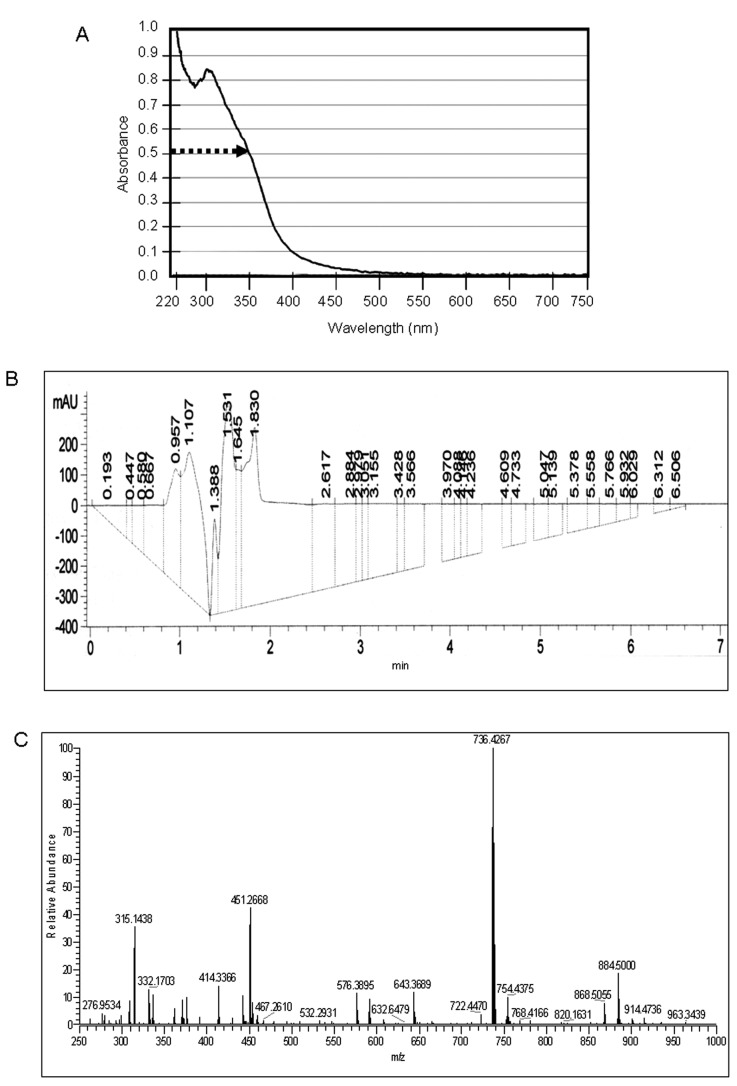
(A) UV spectrum of aqueous berry extract of *Solanum nigrum* (SN). The scan was obtained from 200–750 nm and then subjected to absorbance at 750 nm using gallic acid as standard. (B) HPLC chromatogram of an aqueous SN extract demonstrating the presence of various polyphenolic constituents. (C) Electrospray product ion mass spectra of SN extract. The ion chromatograms were extracted at m/z corresponding to the molecular mass and structure of various polyphenolic compounds were detected. The details are described in the Materials and methods section.

The spectroscopic scanning of aqueous SN derived from the ripe berries demonstrated a high peak at a wavelength of 200–400 nm (Fig. 1A). Analysis of the SN extract revealed that it contains ~28% total polyphenols and this peak corresponds to lipids, steroids, vitamins and flavoproteins. Next we performed an HPLC scan at wavelengths ranging from 200–590 nm to determine the presence of various constituents in the SN extract (Fig. 1B). Altogether, a total of 30 peaks were recorded in the aqueous SN extract during the scanning process, most of which correspond to water soluble phenolics (Fig. 1B). Since the major polyphenols previously identified in SN are catechins and their derivatives, we narrowed the wavelength scan between 335 and 360 nm, which identified five major peaks with retention times of 0.974 (23.4%) 1.101 (33.1%), 1.387 (7.5%), 1.532 (18.9%) and 1.823 min (17.1%) (data not shown). The peaks correspond to gallocatechin, caffeic acid, gallocatechin gallate, rutin and naringenin, respectively. Previous studies have identified several polyphenols in the extracts of leaves, stem and fruits of SN which include gallic acid, protocatechuic acid, epigallocatechin, chlorogenic acid, catechin, gentisic acid, vanillic acid, caffeic acid, syringic acid, epicatechin, epigallocatechin gallate, gallocatechin gallate, p-coumaric acid, ferulic acid, rutin, m-coumaric acid, narigenin, luteolin, myricetin, quercetin, apigenin, kaempferol, and hesperetin together with some anthocyanins (17). The other polyphenolic compounds might be present in the aqueous SN extract although their percentage might vary in our studies. The MS-MS data confirmed the presence of some steroidal glycosides and steroidal alkaloids which includes α-solanine [molecular weight (MW) 867.49798; (M+H) 868.50525], solasodine [MW 413.32935 (M+H) 414.33663], solasonine [MW 883.49289 (M+H) 884.50017], and solamargine [MW 883.49289 (M+H) 884.50017], respectively (Fig. 1C).

**Figure 2:**
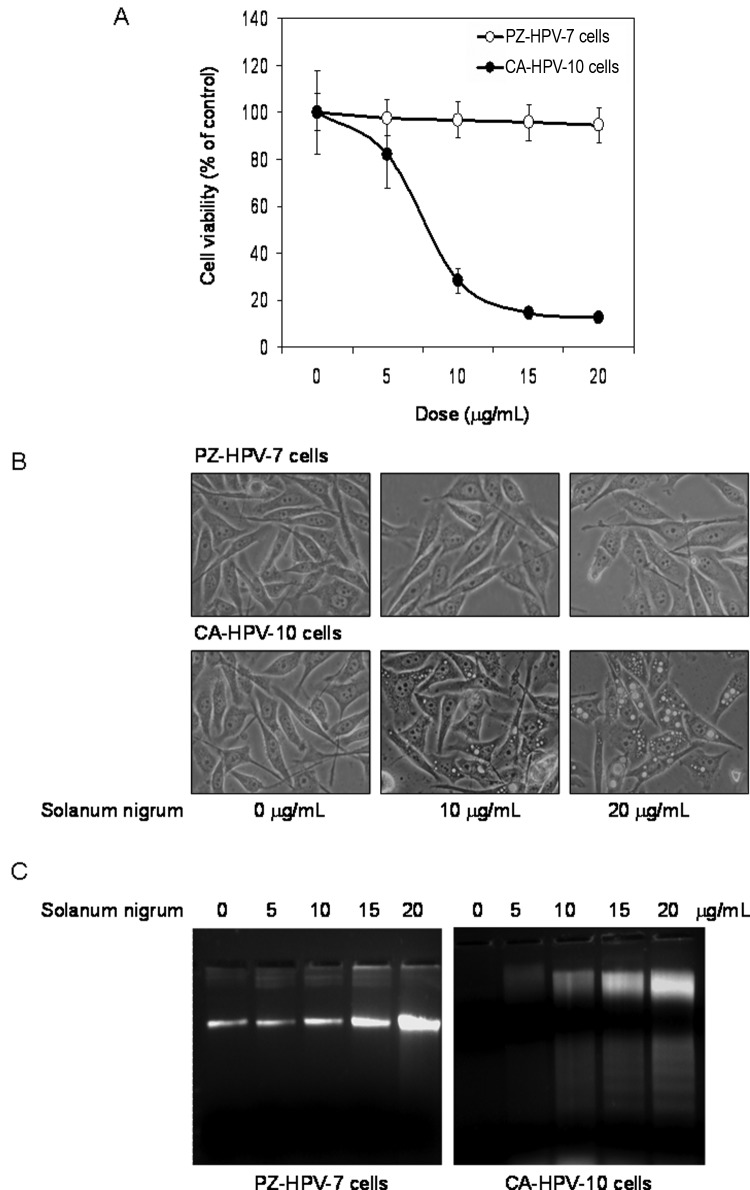
(A) Effect of SN extract on cell viability in non-cancerous PZ-HPV-7 cells, and their cancer counterpart CA-HPV-10 cells. The cells were exposed to the specified concentration of SN extract for 24 h, and viability of the cells was determined by the MTT assay. Cell viabilities are depicted as percentages; vehicle-treated cells were regarded as 100% viable. The data represent the mean of 3 experiments performed in triplicate. (B) Light microscopy images of virally transformed normal human prostate epithelial PZ-HPV-7 cells and their cancer counterpart CA-HPV-10 cells treated with aqueous SN extract. Treatment of CA-HPV-10 cells with SN extract exhibits morphological changes consistent with apoptosis, whereas no such morphological changes were observed in PZ-HPV-7 cells. (C) DNA fragmentation by SN extract in CA-HPV-10 cells. The cells were treated with 5–20 μg/ml concentration of SN and 48 h later, the cells were collected, DNA was isolated and subjected to agarose gel electrophoresis, followed by visualization of bands under UV light. The PZ-HPV-7 cells did not indicate DNA fragmentation by SN extract. The details are described in the Materials and methods section.

Next we evaluated the growth inhibitory response of the aqueous SN extract in virally transformed prostate epithelial PZ-HPV-7 cells and carcinoma CA-HPV-10 cells obtained from the same individual. Exposure of PZ-HPV-7 cells to SN exhibited a modest decrease of 5.5% in cell viability at the highest concentration of 20 μg/ml. In contrast, treatment of prostate cancer CA-HPV-10 cells with 5–20 μg/ml resulted in 17.9–87.3% decrease in cell viability. This demonstrates that cancer cells were more sensitive to SN-mediated loss of cell viability which occurred at much lower doses and was more pronounced than PZ-HPV-7 cells (Fig. 2A). Prominent changes in cell morphology were observed 24 h after treatment with SN extract in prostate cancer CA-HPV-10 cells, which displayed blebbing, loss of cell membrane symmetry and attachment, cell shrinkage, nuclear fragmentation and chromatin condensation when viewed under a light microscope. However, the morphology of normal prostate epithelial PZ-HPV-7 cells remained unchanged after SN treatment (Fig. 2B).

To confirm that reduced cell viability and cell proliferation of prostate cancer CA-HPV-10 cells after treatment with the SN extract was due to cell apoptosis, we performed a DNA fragmentation assay. Our results demonstrate that treatment with 5–20 μg/ml of SN extract for 48 h caused fragmentation of nucleosomal DNA, a characteristic feature of apoptosis, in prostate cancer CA-HPV-10 cells but not in normal prostate epithelial PZ-HPV-7 cells (Fig. 2C).

**Figure 3:**
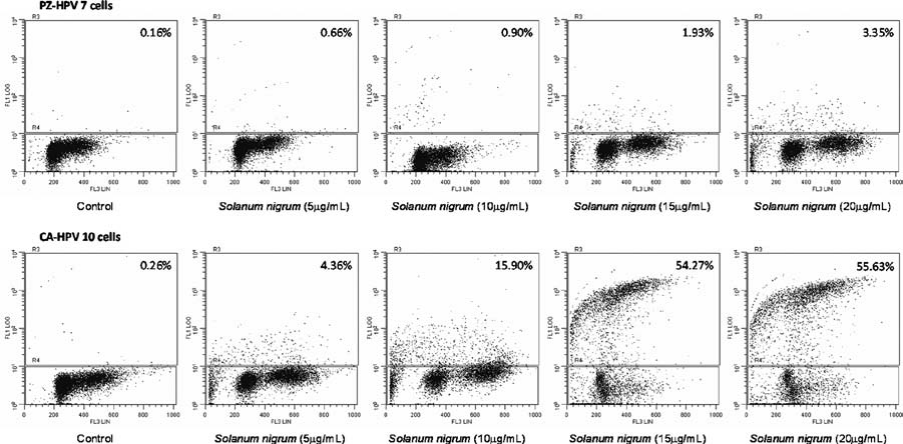
Effect of SN extract on induction of apoptosis in virally-transformed normal human prostate epithelial PZ-HPV-7 cells, and their cancer counterpart CA-HPV-10 cells. The cells were treated with 5–20 μg/ml concentration of SN extract for 48 h and the number of cells undergoing apoptosis was determined using the TUNEL assay. Details are described in the Materials and methods section.

We next quantified the extent of apoptosis by measuring the number of TUNEL-positive cells. The PZ-HPV-7 and CA-HPV-10 cells were treated with 5–20 μg/ml concentration of aqueous SN extract for 48 h. Exposure of cells to SN extract resulted in 4.36% TUNEL-positive cells at 5 μg/ml, 15.90% at 10 μg/ml, 54.27% at 15 μg/ml and 55.63% at 20 μg/ml SN, compared to control (0.26% stained cells) (Fig. 3). In contrast, a modest percentage of TUNEL-positive stained cells were noted in PZ-HPV cells after similar doses of aqueous SN treatment. Compared to the control group where 0.16% cells were TUNEL-positive, exposure to the SN extract caused a modest increase of 3.35% in the percentage of TUNEL-positive cells at the highest concentration of 20 μg/ml in PZ-HPV-7 cells.

**Figure 4:**
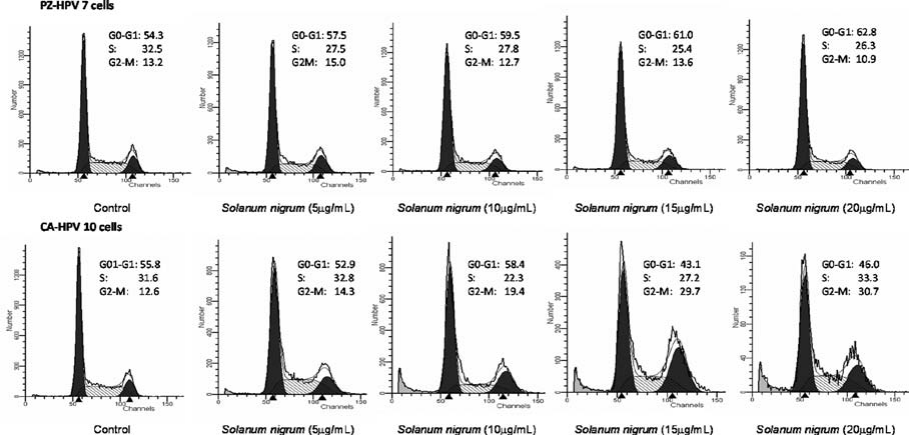
Effect of SN extract on DNA cell cycle in non-cancerous PZ-HPV-7 cells, and their cancer counterpart CA-HPV-10 cells. Log phase growing cells were exposed to increasing concentrations of SN extract (5–20 μg/ml) in complete medium for 24 h, stained with PI (50 mg/ml) and analyzed by flow cytometry. Percentages of cells in subG1, G0/G1, S and G2/M phase were calculated using Cell Quest and ModFit cell cycle analysis software, represented in the right side of the histogram. Data shown here are from a representative experiment repeated three times with similar results. The details are described in the Materials and methods section.

Next we evaluated whether decrease in cell viability and induction of apoptosis in prostate cancer cells by SN is a result of cell cycle deregulation (Fig. 4). Compared to the control group (12.6% in G2/M phase), exposure of prostate cancer CA-HPV-10 cells to the aqueous SN extract resulted in an appreciable arrest of cancer cells in the G2/M phase of the cell cycle after 24 h. The exposure caused an increase accumulation of 14.3% in the G2/M phase at a concentration of 5 μg/ml, an effect that further increased to 19.4% at 10 μg/ml, 29.7% at 15 μg/ml and 30.7% at the highest concentration of 20 μg/ml in CA-HPV-10 cells. In contrast, a modest arrest in the G0/G1 phase was noted in PZ-HPV-7 cells after similar doses of aqueous SN treatment. Compared to the control group in which 54.3% cells were present in the G0/G1 phase, exposure to the SN extract caused an arrest of 62.8% at the highest concentration of 20 μg/ml in PZ-HPV-7 cells (Fig. 4).

**Figure 5:**
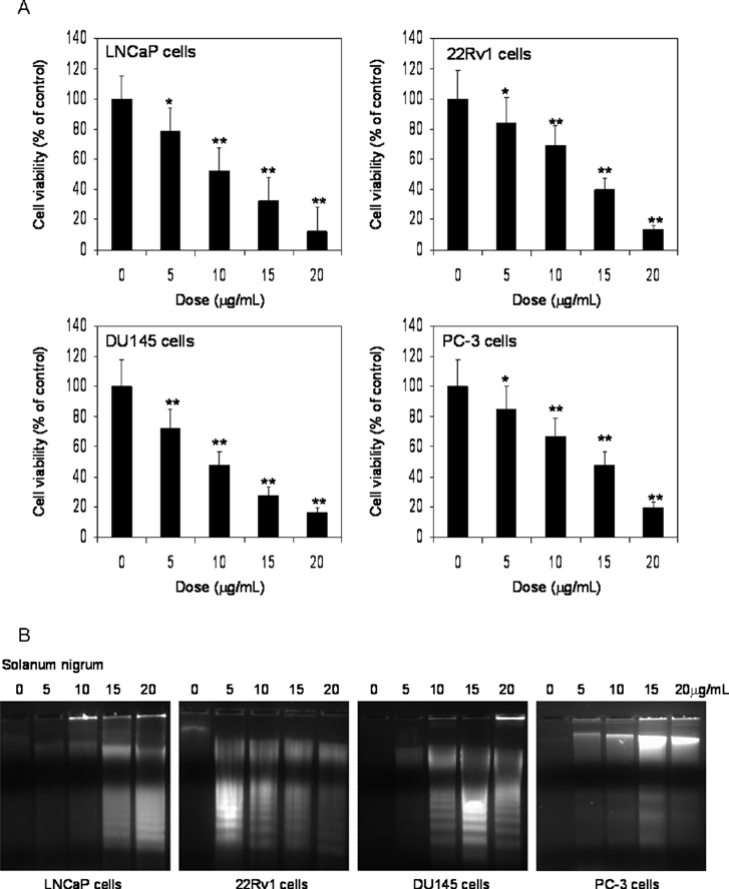
Effect of SN extract on cell viability and induction of apoptosis in human prostate cancer 22Rv1, LNCaP, DU145 and PC-3 cells. (A) The cells were exposed to 5–20 μg/ml concentration of SN extract for 24 h, and viability of the cells was determined by the MTT assay. Cell viabilities are depicted as percentages; vehicle-treated cells were regarded as 100% viable. The values represent mean ± SD of three different assays in duplicate; ^*^P<0.05; ^**^P<0.001, compared to control. (B) DNA fragmentation assay. The cells were treated with vehicle or 5–20 μg/ml concentration of SN extract for 48 h, collected for DNA isolation and subjected to agarose gel electrophoresis, followed by visualization of bands under UV light. All prostate cancer cells underwent DNA fragmentation by the SN extract. Details are described in the Materials and methods section.

Since prostate cancer is known to be intrinsically heterogeneous and represents a mixture of androgen-responsive and refractory cells at the time of clinical diagnosis, we next investigated the cell growth inhibitory and apoptotic response of aqueous SN extract using cultured cells that broadly represent the spectrum of stages of prostate cancer as well as their response to androgens. A panel of four other human prostate cancer cell lines, the LNCaP (androgen-responsive), 22Rv1 (androgen-repressive), DU145 (androgen-refractory), and PC-3 (androgen-refractory) cell lines were used for evaluating cell growth inhibition and apoptotic response with the SN extract. Exposure of all four cancer cell lines with aqueous SN extract at 5–20 μg/ml concentration resulted in significant decreases in cell viability ranging from 12–88% and resulted in apoptosis in all the cell lines (Fig. 5). This decrease in cell viability and the apoptotic response of SN extract was not limited to prostate cancer CA-HPV-10 cells as similar treatment resulted in growth inhibition and apoptotic cell death in all the types of prostate carcinoma cells independent of their disease stage and androgen association.

## Discussion

Chemotherapy is one of the major treatment strategies to eliminate residual cancer cells and prevent metastasis after surgery or radiotherapy (18). Chemotherapeutic efficacy or therapeutic index is contingent upon differences during sensitivity between tumor and normal tissue. The chemotherapeutic indices of known chemotherapeutic agents are often small, with few differences between the doses required for optimal anti-tumor activity which produces severe toxicity to normal tissues (19). The ideal anticancer agent is expected to exert minimal adverse effects to normal tissue and a maximum ability to sacrifice tumor cells and/or inhibit tumor growth. Our studies, for the first time, demonstrate that aqueous polyphenolic rich SN extract possesses maximum anti-tumor effect with minimal toxicity to non-cancerous cells and could potentially play an important role in the therapy or perhaps in the prevention of prostate cancer.

A number of biological pathways can be exploited in the prevention and/or therapy of cancer (20,21). Apoptosis is a physiological process for the elimination of redundant or damaged cells. It is a mode of cell death in which individual cells are deleted from tissue during normal tissue turnover and is characterized by morphological changes including cell shrinkage, membrane blebbing, chromatin condensation, and DNA fragmentation (22). In cancer, resistance to apoptosis affords tumor cells a survival advantage and the ability to be sustained and to proliferate. Apoptosis induction remains arguably the most potent defense against cancer progression as most of the current used chemotherapeutic drugs inhibit cancer cell proliferation by inducing apoptosis (23,24). Unfortunately, chemotherapeutic agents are not designed to selectively target cancer cells. Consequently, chemotherapeutic agents typically damage normal cells, an adverse effect that affects therapeutic modality. Therefore, agents capable of preferably eliminating cancer cells without affecting normal cells offer the safest modality for cancer treatment. Our studies demonstrate a striking difference in the induction of apoptosis after treatment with aqueous SN extract in various human prostate cancer cells representing various disease stages as well as androgen status, compared to non-cancerous cells. These studies further strengthen our findings that the aqueous SN extract could be an effective agent for the treatment of various stages of prostate cancer. Further studies are needed to determine the specific pathways through which the SN extract induces apoptosis in prostate cancer cells.

Uncontrolled cellular proliferation is a hallmark of all cancer, and the blockade of the cell cycle is regarded as an effective strategy for eliminating cancer cells (25). In recent years, many chemotherapeutic agents have been shown to impart anti-proliferative effects via arrest of cell division at certain checkpoints in the cell cycle (24,25). The concept of cell cycle-mediated apoptosis has gained increasing attention as this pathway may provide minimal opportunity for acquired drug resistance, decreased mutagenesis and reduced toxicity (23–25). Our findings on cell cycle analysis indicate that the aqueous SN extract has the ability to induce G2/M phase arrest in prostate cancer CA-HPV-10 cells, indicating that the cells were arrested at the mitotic stage. It will be tempting to decipher which cell cycle regulatory molecules are affected by SN in prostate cancer cells.

The aqueous extract of SN was observed to be the most effective in causing cytotoxicity to various prostate cancer cells and a minimal effect on non-cancerous cells. The ripe berries of *Solanum nigrum* contain many steroidal glycosides, steroidal alkaloids and steroidal oligoglycosides, which include solamargine, solasonine, solavilline, solasodamine, solasodine and solanine (26,27). The fruit is rich in polyphenolic compounds including gallic acid, protocatechuic acid, catechins, caffeic acid, epicatechin, rutin and naringenin (28). It is used in traditional folk medicine and shown to possess anti-inflammatory, antipyretic and antioxidant activity (29). A previous study investigated the effect of SN on skin cancer cells, which resulted in inhibition in cell migration and invasion (30). Recent studies demonstrate the anti-proliferative activity of SN in some type of human cancer cells (11–14,30,31). Treatment of human breast cancer MCF-7 and AU565 cells with extracts of fruit and leaves of SN inhibited cell growth and induced apoptosis and autophagy in these cell lines (32). A 150 kDa glycoprotein isolated from *Solanum nigrum* L. has been reported to modulate the DNA-binding activities of the transcription factors NF-κB and AP-1 and activates the mitochondrial apoptotic pathway in hepatocellular carcinoma, breast, cervical, and colorectal cancer cells (33–36). This protein has reported to have a stimulatory effect on the release of mitochondrial cytochrome c, cleavage of pro-caspase-9, −3, and poly (ADP-ribose) polymerase resulting in apoptosis (36). In *in vivo* studies using the aqueous SN extract, inhibition of metastasis in a mouse B16-F1 melanoma xenograft and a lung metastasis model were observed (30). It is suggested that the pronounced anti-proliferative activity of the SN extract on various human prostate cancer cells observed in the present study is critical to uncover the target proteins and to determine the molecular pathways by which SN mediates cancer-specific effects.

In conclusion, for the first time we demonstrate the selective effects of the aqueous SN extract in causing cell cycle arrest and apoptosis in various human prostate cancer cells with minimal effects on normal cells. Our results also show that SN may be an effective herb for the treatment and/or prevention of prostate cancer. Further studies are needed to verify our data and evaluate its effectiveness in preclinical models of prostate cancer. Moreover, an additional study is needed to separate its bioactive components and to evaluate whether the whole mixture or an individual constituent is worthy of further drug development.

## Abbreviations

DNA: deoxyribonucleic acid
PARP: poly (ADP-ribose) polymerase
HPLC: high performance liquid chromatography
SN: *Solanum nigrum*
